# Massive dermal ulcerative lesions because of brown recluse spider bite: a rare case report and review of literature

**DOI:** 10.1093/jscr/rjad357

**Published:** 2023-06-21

**Authors:** Mahdi Fakhar, Shahriar Alian, Ashkan Zakariaei, Fatemeh Nourzad, Zakaria Zakariaei

**Affiliations:** Iranian National Registry Center for Lophomoniasis and Toxoplasmosis, Mazandaran University of Medical Sciences, Sari, Iran; Antimicrobial Resistance Research Center, Department of Infectious Diseases, Mazandaran University of Medical Sciences, Sari, Iran; Student Research Committee, Babol Branch, Islamic Azad University, Babol, Iran; Toxicology Ward, Qaemshahr Razi Hospital, Mazandaran University of Medical Sciences, Sari, Iran; Toxicology and Forensic Medicine Division, Mazandaran Registry Center for Opioids Poisoning, Antimicrobial Resistance Research Center, Imam Khomeini Hospital, Mazandaran University of Medical Sciences, Sari, Iran

**Keywords:** Brown recluse spider, Skin lesion, Blisters, Necrotizing fasciitis

## Abstract

A brown recluse spider (BRS) bite is challenging to confirm, but can be clinically diagnosed by considering the location, the season of the year and the clinical manifestations. We described a 26-year-old male who presented after a BRS bite with a skin lesion, bruising, severe swelling and diffuse blisters on the right lower extremity after 3 days. This case should be considered in the differential diagnosis of necrotizing fasciitis. Although spider bite poisoning is rare, proper diagnosis and management are important because, in some cases, it can have devastating outcomes.

## INTRODUCTION

*Loxosceles* spiders, also known as brown spiders, can cause loxoscelism when they bite humans. This condition is characterized by dermonecrotic lesions on the skin and, in severe cases, by systemic changes such as hemolysis, hemolytic anemia, thrombocytopenia and acute kidney injury. Although rare, these cases can be fatal [1, 2]. The brown recluse spider (BRS) is found in temperate and tropical regions worldwide, including northern Iran. These spiders typically hide in dark, enclosed spaces such as behind boxes, rocks or leaves in dry and warm areas [3, 4]. The clinical presentation of a BRS bite typically begins with a small blister in the center, surrounded by erythema and purpura. Within 48–72 h, a necrotic ulcer with a prominent eschar may develop [5, 6]. We report a case of a patient presenting to the emergency department (ED) with widespread cutaneous manifestations, including a dermonecrotic lesion, bruises and blisters, resulting from a BRS bite.

## CASE PRESENTATION

On 10 August 2022, a 26-year-old male with a history of oral opium addiction presented to the ED of a hospital in northern Iran. He complained of a painful skin lesion on his right lower extremity, extensive bruising, blisters and diffused pitting edema from his right dorsal pedis surface to his buttock region. The patient reported being bitten by an insect on his right leg while working on his farm 3 days prior, which resulted in immediate pruritus, redness and mild swelling. Traditional remedies were applied to the initial wound, but the symptoms worsened over time, resulting in diffused bruises and blisters.

The day after the insect bite, a painful, red blister developed at the site. The pain was severe and stabbing. The blister ruptured the next day and progressed into a black lesion. The patient denied any history of travel or systemic symptoms and reported no prior trauma to the affected area. They also denied any history of other illnesses or use of antithrombotic or antiplatelet therapy.

**Figure 1:**
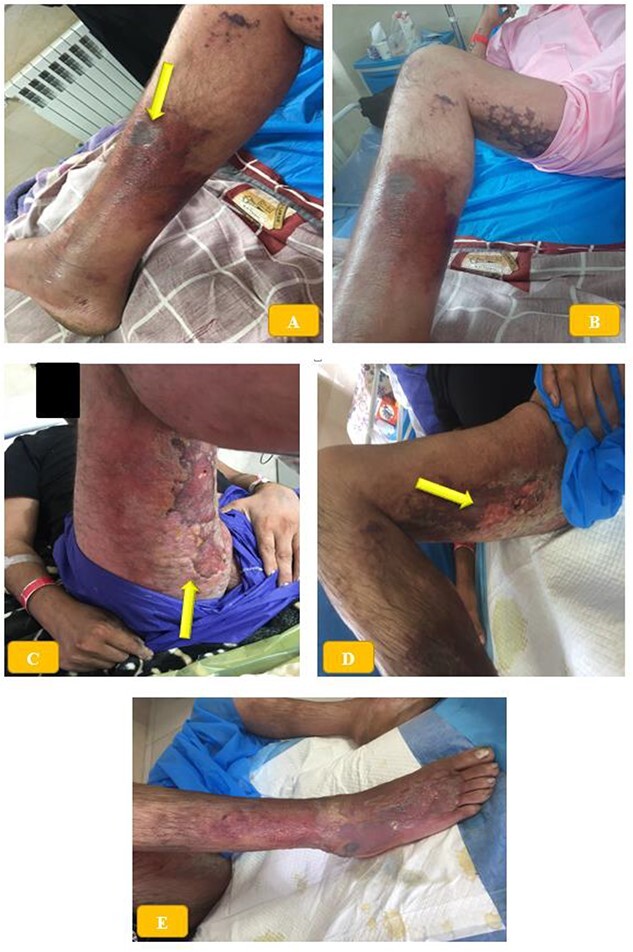
(**A**, **B**) Necrotic skin wound on the medial aspect of the right lower third of the leg, associated with erythema, swelling and warmth extended to the dorsum of the foot. (**C**–**E**) Diffuse bruises and blisters in posterior thigh and buttock region.

The patient was conscious and had no systemic symptoms such as fever, chills, myalgia, sweating, nausea or vomiting. However, he experienced pain and limited mobility in his right lower limb. The patient had a necrotic wound on the medial aspect of his right middle third leg, measuring 3 × 5 cm, with swelling, erythema and warmth that extended to the dorsum of his foot (Fig. 1A and B). The area was tender to palpation. The initial differential diagnosis included cellulitis, necrotizing fasciitis and BRS bite. The patient’s vital signs were recorded as follows: blood pressure of 125/80 mmHg, heart rate of 85 beats per minute, respiratory rate of 16 breaths per minute and temperature of 37.2°C. Laboratory tests conducted during the hospitalization are summarized in Table 1.

**Table 1:** Baseline laboratory results.

Parameter	Initial value	Reference value	Unit
Na	134	135–145	mEq/L
K	4.2	3.5–5	mEq/L
BUN	24	3–23	mg/dL
Cr	1.4	0.6–1.2	mg/dL
Ca	9.1	8.5–10.5	mg/dL
Mg	1.9	1.7–2.2	mg/dL
P	3.3	2.5–4.5	mg/dL
FBS	84	70–110	mg/dL
AST	35	10–40	IU/L
ALT	45	˂45	IU/L
ALP	300	80–306	IU/L
CPK	713	21–232	IU/L
Hb	13	12–16	gr/dL
WBC	19 × 10^3^	4000–10 000	mm^3^
Plt	146 × 10^3^	145 000–450 000	mm^3^
PT	15.4	11–13	s
PTT	34	19–36	s
INR	1.5	1–1.3	Ratio

*Abbreviations:* FBS, fasting blood sugar; Cr, creatinine; BUN, blood urea nitrogen; Hb, hemoglobin; CPK, creatine phosphokinase; AST, aspartate aminotransferase; ALT, alanine aminotransferase; ALP, alkaline phosphatase.

To rule out fracture or dislocation, radiography was performed on the patient’s right lower extremity, which showed diffuse subcutaneous swelling with fat stranding, but no significant findings. Color Doppler ultrasound of the lower limbs did not reveal any deep or superficial vein thrombosis, only soft-tissue swelling. The patient was admitted to the poisoning service and a consultation with infectious disease service was requested.

Considering the patient’s immunization status, human tetanus immunoglobulin and toxoid were prescribed. The wound was washed daily, and broad-spectrum antibiotics were administered (intravenous ciprofloxacin vial 200 mg every 12 h, imipenem 500 mg every 6 h and linezolid 600 mg daily) to prevent necrotizing fasciitis. Corticosteroids, acetaminophen and meperidine were given to manage pain and inflammation. General surgery was consulted for possible wound debridement and recommended local wound care in addition to antibiotics. After 1 week of treatment, the patient showed improvement with reduced pain and swelling. The patient was discharged from the hospital in good condition after 12 days of treatment (Fig. 1C–E). Written informed consent was obtained from the patient for the publication of this report. This study was conducted according to the Declaration of Helsinki principles.

## DISCUSSION

The severity of loxoscelism depends on multiple factors, including the amount and type of poison injected (degree of sphingomyelinase D activity), the gender of the spider (females have more toxic venom), the bite location (central areas of the body) and the host’s response to the venom [7]. BRS bites can cause intravascular and extravascular hemolysis, pulmonary edema and, in rare cases, death [8, 9].

Treatment for BRS bites typically involves systemic antibiotics and local wound care, although more severe cases may require hyperbaric oxygen therapy (HBOT) and skin grafting [10]. HBOT has been used in several cases of loxoscelism a few weeks after the bite and is recommended for nonhealing wounds because of the toxic effects of oxygen against anaerobic bacteria and increased activity of certain antibiotics [11]. However, there is no standardized treatment for BRS bites. Over the years, many treatment options, such as antihistamines, analgesics, antibiotics, corticosteroids, dapsone, colchicine, antivenom, surgical debridement, HBO, electric shock therapy, plasmapheresis and observation, have been recommended for spider bites [12].

Longner *et al*. reported a case of a 16-year-old boy who presented with a necrotic skin lesion believed to be from a spider bite. Despite developing systemic loxoscelism, the patient showed improvement after treatment with steroids, plasmapheresis and intravenous immunoglobulins [12]. Ferreira *et al*. reported a case of a 32-year-old man who was bitten by a brown spider on the lower lip during the COVID-19 pandemic in southern Brazil. The patient developed renal failure, a systemic inflammatory reaction, and pulmonary complications because of both loxoscelism and COVID-19. Unfortunately, the patient died as a result of these complications [13]. Cachia *et al*. reported a young female from Malta who developed localized erythema and pain on her left thigh after a spider bite. The area developed dermonecrosis, systemic symptoms and a generalized erythematous eruption over a few days [14].

## CONCLUSION

While spider bite poisoning is rare, proper diagnosis and management are crucial as it can have devastating consequences in some cases. Obtaining a detailed history from the patient is essential in determining whether a spider bite is the underlying cause of the symptoms.

## CONFLICT OF INTEREST STATEMENT

None declared.

## FUNDING

None.

## AUTHORS’ CONTRIBUTIONS

Z.Z., S.A. and F.N. were involved in the collecting of samples and data. M.F., Z.Z. and A.Z. interpreted the data, wrote and edited the manuscript. A.Z. prepared the draft and submitted the manuscript. All authors reviewed and approved the final version of the manuscript.

## DATA AVAILABILITY

The data are available from the corresponding author and can be obtained upon request.

## INFORMED CONSENT

Written informed consent was obtained from the patient for publication of this case report and any accompanying images. A copy of the written consent is available for review upon request from the journal.

## ETHICS APPROVAL

This study has received written consent from the local ethics committee.
